# The effect of velocity-based strength training on lower limb maximal strength, power, and muscle thickness: a comparative study of sex-specific adaptations

**DOI:** 10.3389/fphys.2026.1787790

**Published:** 2026-05-29

**Authors:** Longkang Guo, Lintao Suo, Wendong Xue, Jingyuan Yang, Xuehaiyue Lv, Haonan Qi, Mushuai Hao, Liang Zhao, Xiaofu Yang, Wei Han

**Affiliations:** 1 Graduate Education College, Shandong Sport University, Shandong, China; 2 School of Physical Education, Hebei Normal University, Shijiazhuang, China; 3 College of Competitive Sports, Shandong Sport University, Rizhao, China

**Keywords:** athletic performance, lower limb strength performance, sex differences, velocity loss, velocity-based strength training

## Abstract

**Purpose:**

This study aims to explore the effects of Velocity-Based Strength Training (VBT) on lower-limb strength, power, and muscle thickness in athletes of different sexes. It aims to identify sex-specific differences in training adaptations and explore how sex modulates training outcomes.

**Methods:**

A total of 28 participants with more than two years of systematic resistance training experience were recruited and divided into male and female groups according to sex, with 14 participants in each group. Four participants dropped out throughout the 8-week intervention, leaving a final sample of 12 males and 12 females. All participants completed an 8-week barbell back squat training program based on a VBT protocol. The training was performed thrice a week, with the load intensity progressively increasing from 65% to 75% of one-repetition maximum (1RM), and the training volume was set at 20% velocity loss (VL). Before and after the training intervention, measurements were taken for the following indicators: squat 1RM, jumping ability [countermovement jump (CMJ), squat jump (SJ), standing long jump (SLJ)], 30-meter sprint performance (T30), and rectus femoris thickness (RFT).

**Results:**

After the VBT intervention, both groups exhibited significant improvements in lower limb maximal strength, jump performance, sprint ability, and rectus femoris thickness (*P* < 0.05). However, differences in training effects were observed between the two groups: the male group showed greater improvement magnitudes in squat 1RM (*Hedges’ g* = 1.52, growth rate = 23.46%, *P* < 0.001), CMJ (*Hedges’ g* = 1.19, growth rate = 17.70%, *P* < 0.001), SJ (*Hedges’ g* = 1.28, growth rate = 17.93%, *P* < 0.001), and SLJ (*Hedges’ g* = 0.63, growth rate = 5.00%, *P* < 0.001); the female group demonstrated a more prominent improvement in T30 (*Hedges’ g* = 1.95, growth rate = 17.41%, *P* < 0.001). RFT scores significantly increased in both groups, with no significant difference between the two groups (*P* > 0.05).

**Conclusion:**

VBT can significantly improve lower-limb strength, power, and muscle thickness in athletes of both sexes. However, training effects vary by sex: males show greater improvements in maximal strength and jump performance, while females demonstrate more prominent sprint gains.

## Graphical Abstract

**Figure:**
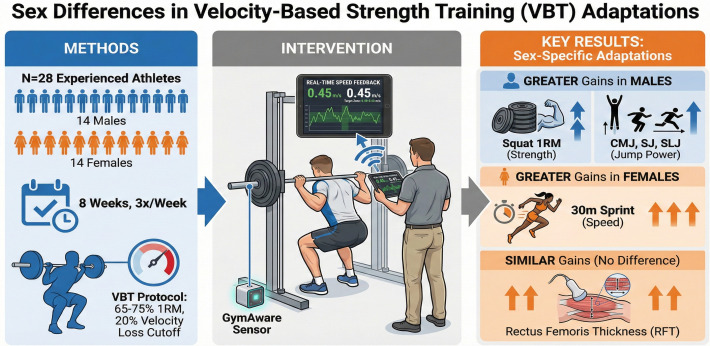

## 1 Introduction

Numerous studies have confirmed that resistance training exerts profound effects on muscle hypertrophy, maximal strength enhancement, increasing the rate of force development, and improving adaptations related to force output (Behm and Sale, 1993; Dorrell et al., 2020). In traditional resistance training, coaches typically prescribe resistance training loads based on the percentage of one-repetition maximum (%1RM), coupled with a predetermined number of sets and repetitions (Weakley et al., 2017), This method is referred to as 1RM percentage-based training (PBT). However, athletes’ training status or performance is constantly changing due to various factors, such as circadian rhythm, stress, training-induced fatigue, nutrient intake, and sleep quality, which can result in fluctuations in 1RM of up to 36% (Flanagan and Jovanovic, 2014; Zourdos et al., 2016). Therefore, setting training intensity based on a previously measured 1RM may result in a discrepancy between the target intensity and an individual’s actual functional capacity, thereby reducing training effectiveness and even potentially resulting in diminished training outcomes (Padulo et al., 2012). In addition, Individuals demonstrate substantial variability in their capacity to complete repetitions at the same %1RM (Shimano et al., 2006), Training of the same intensity may lead to insufficient stimulation for some trainees while causing excessive fatigue for others.

In recent years, a growing body of research has indicated that in resistance training, setting target velocity zones to control load intensity and using the percentage of velocity loss (VL)—defined as the percentage of velocity loss relative to the peak velocity—to monitor fatigue levels and training volume constitutes an effective method for training load monitoring (Sanchez-Medina and Jose Gonzalez-Badillo, 2011). This method is termed Velocity-Based Training (VBT), which leverages the strong correlations between velocity, %1RM, movement repetitions, and fatigue to design, monitor, and adjust resistance training loads (Gonzalez-Badillo et al., 2011; SIndiani et al., 2020). Compared with %1RM training PBT, VBT enables more precise control of training loads, is less affected by individual physiological fluctuations, and achieves similar or even superior improvements in athletic performance with a lower level of fatigue (Dorrell et al., 2020). In addition, due to the strong correlation between the percentage of VL and the number of repetitions, VBT uses VL instead of fixed repetitions to control the level of fatigue per set. This approach not only prevents overtraining-induced fatigue but also achieves a comparable level of training stimulus (Gonzalez-Badillo and Sanchez-Medina, 2010).

Sex, as a key biological variable, has long been insufficiently emphasized in physical training research, and its importance has only gradually become prominent in recent years. The mechanism underlying sex differences involves specific variations at the neuromuscular, endocrine, and anatomical-physiological levels (Handelsman, 2017; Hunter and Senefeld, 2024). The testosterone-mediated physiological effects are central to understanding sex differences: the testosterone level in adult males is approximately 15 times that in females. This significant difference promotes an increase in the proportion of fast-twitch muscle fibers (TypeII) in male skeletal muscle, an expansion of muscle fiber cross-sectional area, and regulates muscle protein synthesis through androgen receptors (Handelsman, 2017; Clayburn et al., 2020; Senefeld et al., 2020; Hunter et al., 2023). In terms of athletic performance, although females have relative disadvantages in absolute strength and power, they possess higher fatigue resistance and oxidative metabolism efficiency, demonstrating unique adaptive characteristics in muscle endurance and hypertrophy-related indicators (Ansdell et al., 2019; Pareja-Blanco et al., 2020). However, in traditional resistance training research, controversies remain regarding the training effects across different sexes groups, with some studies indicating no significant differences in training outcomes between males and females (Roberts et al., 2020; Rissanen et al., 2022), In contrast, other studies argue that males exhibit superior training outcomes compared to females (Ramirez-Campillo et al., 2016; Cardoso de Araujo et al., 2020). This inconsistency primarily stems from limitations in study design. Most studies have focused on untrained individuals, and there are significant differences in baseline strength levels between male and female participants, making “strength level” a key confounding variable. Given the aforementioned issues, the adaptive effects of resistance training in athletes of different sex with the same relative strength level warrant further in-depth investigation.

Therefore, the present study focuses on VBT and aims to explore the training effects of this model on lower-limb strength, power, and muscle thickness in athletes of different sexes with the same relative strength level under identical load intensity and VL thresholds. It further enriches the research findings related to sex-specific adaptations in the field of VBT, providing practical and theoretically grounded training recommendations for coaches, practitioners, and researchers.

## 2 Method

### 2.1 Subject

To ensure the reliability of the study results, an *a priori* sample size calculation was performed using G*Power (version 3.1.9.2, Germany). The statistical test selected was “ANOVA: Repeated measures, within–between interaction,” with the parameters set as f = 0.3, 1 − β = 0.80, and α = 0.05 (Rossi et al., 2024). The calculation indicated that at least 24 participants were required to effectively assess the time × group interaction effect. To account for potential participant attrition during the study, a total of 28 collegiate athletes from Shandong Sport University were ultimately enrolled in this study. The inclusion criteria for participants were as follows: at least 2–3 years of squat training experience, no history of cardiovascular disease, neuromuscular disorders, or lower extremity sports-related injuries, and proficiency in performing barbell back squats with external resistance. The 28 participants were divided into two groups by sex: the Male (n=14) and the Female (n=14). During the 8-week intervention, two participants from the male group and two from the female group withdrew for personal reasons. A total of 24 participants were therefore included in the final statistical analysis, with 12 participants in the male group and 12 in the female group. As the number of dropouts was the same in both groups, and all withdrawals were due to personal reasons unrelated to the intervention or outcome assessment, attrition was considered random. It showed no clear imbalance between groups and was unlikely to cause systematic bias in the main findings. To ensure that the two groups had comparable relative strength levels, an independent-samples *t*-test was used to compare the baseline ratio of squat strength to body mass between groups. The results showed no significant difference in baseline relative strength between the male and female groups (*P* > 0.05), indicating that the two groups were comparable. The detailed characteristics of the participants are presented in **Table 1**. This study was approved by the Ethics Committee of Sports Science at Shandong Sport University (Approval No.: 2022030) and adhered to the Declaration of Helsinki. None of the participants used any drugs, medications, or dietary supplements known to potentially modulate physical performance, in strict adherence to the ethical principles of the Declaration of Helsinki.

**Table 1:** Basic information of subjects.

Group	Age(years)	Height (cm)	Body mass (kg)	1 RM/body mass	Resistance training experience (years)
Male	22.09 ± 1.22	177.84 ± 6.79	82.67 ± 16.86	1.35 ± 0.20	2.68 ± 0.71
Female	19.50 ± 0.90	166.43 ± 4.55	60.50 ± 5.37	1.33 ± 0.18	2.32 ± 0.80

### 2.2 Experimental procedure and control

This study adopted a longitudinal experimental design to investigate the long-term effects of VBT on lower-limb strength, jumping ability, sprint performance, and muscle thickness in athletes of different sexes with the same relative strength level. The total study duration was 12 weeks, comprising a 2-week familiarization period, an 8-week intervention period, and pre- and post-intervention testing phases (2 weeks combined). Participants trained 3 times per week with a 48-hour rest interval between training sessions (Zhang et al., 2024). A total of 24 training sessions were completed throughout the intervention period. During the intervention period, participants were instructed to adhere to standardized dietary and sleep protocols, and female participants were required to refrain from using oral contraceptives throughout the study. The training exercise was the back squat, and all groups were trained with the same relative load, training volume, visual feedback, and motivational strategies. Sex was treated as the independent variable. During the experiment, if a participant’s mean velocity (MV) deviated from the target range by ±0.04 m/s (either below or above), the load intensity was adjusted promptly to ensure MV remained within the specified velocity zone. Training sets were terminated immediately when a participant’s VL exceeded the predetermined threshold. All participants were assessed three times before and after the 8-week training intervention. The assessments were conducted over three days, with a 48-h interval between testing sessions (Zhang et al., 2024). For female participants, pre- and post-intervention assessments were scheduled outside the menstrual bleeding phase to reduce the potential influence of menstruation on performance outcomes. All tests were conducted in a laboratory setting under the direct supervision of researchers. To ensure consistency, each participant completed the tests at the same time of day (± 1 hour) and under controlled environmental conditions (approximately 26 °C; relative humidity: 68%). The specific test contents were as follows: On Day 1, the tests included the squat jump (SJ) and countermovement jump (CMJ); on Day 2, the standing long jump (SLJ) and 30-meter sprint(T30); on Day 3, the tests included (rectus femoris thickness RFT) and 1RM. All participants maintained the same warm-up protocol and test sequence in both the pre-intervention and post-intervention assessments.

### 2.3 Procedure

#### 2.3.1 Familiarization period and standardized warm-up

During the 2-week familiarization period prior to the formal experiment, participants were fully briefed on the experimental procedures, test items, and precautions. Two familiarization training sessions were arranged to provide technical guidance on the barbell back squat, ensuring all participants mastered the correct movement technique before undergoing the first assessment. The back squat movement standards referenced the “back squat” technical guidelines established by the National Strength and Conditioning Association (NSCA) (Waller et al., 2009). Participants were instructed to place the barbell across the posterior deltoids and the upper trapezius (base of the neck), with feet shoulder-width apart or slightly wider and toes pointing slightly outward. After maintaining a stable initial posture, they bent at the hips to squat until the top of the thighs was parallel to the floor, followed by a rapid ascent. The same barbell squat rack was used throughout the study to ensure test consistency. Prior to each test and training session, participants performed a standardized warm-up protocol, which included myofascial release, static and dynamic stretching, crawling drills, and jogging, and barbell back squats with a 20-kg load (2 sets of 10 repetitions per set). Additionally, all tests were conducted under the careful supervision and spotting of the same researcher, aiming to prevent potential injuries caused by participants’unfamiliarity with the training methods.

#### 2.3.2 Strength assessment

**Figure 1:**
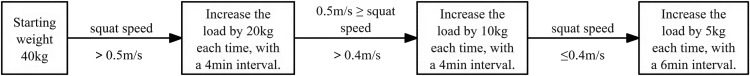
Load-speed test with weight increments.

In this study, a reliable and validated linear position transducer (GymAware RS; Kinetic Performance Technologies, Canberra, Australia) was used to conduct the squat 1RM test (Zangakis et al., 2019). The MV of each repetition performed by the participants was recorded via the accompanying tablet software of the transducer. The squat 1RM testing protocol was as follows: an initial load of 40 kg was applied. If the participant’s MV during the squat exceeded 0.5 m/s, the load was increased by 20 kg for the next set, with a 4-minute rest interval between sets. When the MV ranged between 0.4 m/s and 0.5 m/s (inclusive of 0.5 m/s and exclusive of 0.4 m/s), the load was increased by 10 kg for the subsequent set, with a 4-minute inter-set rest. Once the squat MV dropped to ≤ 0.4 m/s, the load was increased by 5 kg for the next set, and the inter-set rest interval was extended to 6 minutes. This process continued until the participant was unable to complete a successful squat. The load of the last successfully completed squat was defined as the participant’s squat 1RM (**Figure 1**).

#### 2.3.3 Jumping ability test

These included the SJ, CMJ, and SLJ. All tests were conducted following a standardized warm-up and two practice jumps to ensure participants were familiar with the testing movements. The SJ and CMJ tests were performed using the My Jump 2 app, which has been proven to have high accuracy in previous studies (Brooks et al., 2018). For the SJ test, participants were required to place their hands on their hips, start from a position where the knees were approximately at 90°, and jump vertically upward as forcefully and quickly as possible, with the lower limbs avoiding any plyometric movements of any amplitude. For the CMJ test, participants also placed their hands on their hips, squatted to their personal optimal depth at a self-selected speed, and then jumped vertically upward with maximum effort using both feet. For the SLJ test, participants were instructed to stand with their feet shoulder-width apart or slightly narrower, knees slightly flexed, and arms swinging naturally back and forth. During the forward swing of the arms, they extended their legs; during the backward swing, they bent their knees downward. At takeoff, they pushed off rapidly forward with both feet and exerted active force, fully extending the knees while driving the hips forward. After passing the highest point of the jump, they actively bent their knees and contracted their abdomen, landing on their heels first, followed by knee flexion for buffering and leaning the upper body forward. Subsequently, a steel tape measure was used to measure the distance from the starting line (i.e., the tips of the toes) to the landing point of the heels, which was recorded as the SLJ distance. All indicators were tested twice, with a 2-minute rest interval between each test, and the best score from the two attempts was recorded.During the pre-intervention testing of all participants, the test-retest reliability of the jump performance assessments was evaluated. The results showed that for the SJ, the intraclass correlation coefficient (ICC) was 0.97 (95% confidence interval [CI]: 0.95–0.99) with a coefficient of variation (CV) of 3.1%; for the SLJ, the ICC was 0.96 (95% CI: 0.93–0.98) with a CV of 2.8%; and for the CMJ, the ICC was 0.97 (95% CI: 0.94–0.98) with a CV of 1.82%. These findings indicate that the tests have high reliability and stability.

#### 2.3.4 Sprint ability test

The short-distance sprint ability test was conducted on an outdoor track using a four-gate infrared timing and sensitivity testing system (Haugen et al., 2014). Participants were required to complete two T30 with a three-minute rest interval between attempts. To eliminate differences in reaction time at the start between male and female athletes, pure photoelectric gate-triggered timing was adopted: timing commenced when the starting photoelectric gate was blocked and terminated when the finishing photoelectric gate was blocked, with only the movement time of athletes passing through the test section recorded. After participants crossed the 30-meter finish line, the test results were documented by staff via the display screen. Participants were instructed to sprint at maximum effort, and the best performance from the two attempts was finally recorded. The timing for all tests was operated by professional referees to ensure the consistency and accuracy of the testing process. This test demonstrated good reliability, with a test-retest ICC of 0.90 (95% CI: 0.82–0.95) and a CV of 2.17%.

#### 2.3.5 Rectus femoris thickness testing

The rectus femoris thickness was measured using a musculoskeletal ultrasound device (VINNO 5, Vinno Technology Co., Ltd., Kunshan, Jiangsu Province, China) with a 20 Hz probe. For the test, participants were placed in a supine position with their bodies relaxed. The tester applied ultrasonic coupling gel to the tip of the probe and positioned the probe perpendicular to the surface of the rectus femoris for measurement. To ensure the consistency of the measurement position, a meter stick and a protractor were used to record the muscle measurement site after each test, enabling accurate repositioning during the post-intervention test and thus improving test accuracy. Each participant underwent two measurements; if the error between the two measurements exceeded 5%, a third measurement was performed until the error was less than 5%, at which point the measurement data were deemed valid. When recording the rectus femoris thickness, the distance from the upper edge of the patellar joint to the measured muscle and the angle between the probe and the vertical line from the patellar joint to the muscle must be clearly documented. The test-retest reliability of the rectus femoris thickness measurement showed an ICC of 0.89 (95% CI: 0.81–0.94) and a CV of 1.99%, indicating that this measurement method has high reliability and stability.

### 2.4 Training protocol

**Figure 2:**
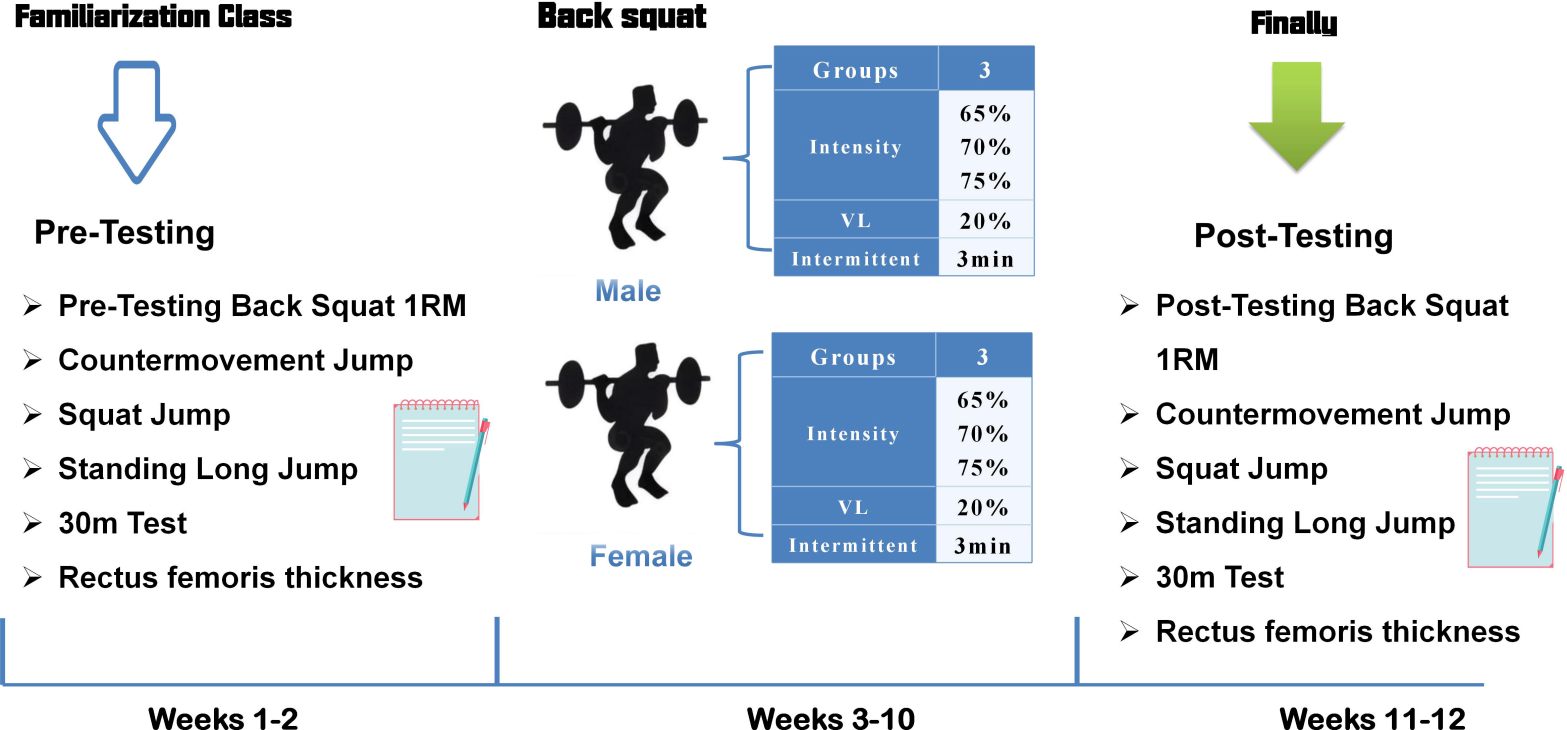
Experimental test flow chart.

During the intervention period, participants were prohibited from engaging in any technical, tactical, or general motor skill training, nor did they participate in any competitions, to avoid interference from other factors on the experimental results. The same researchers were involved in all training intervention sessions to ensure the consistency of the experimental process and the accuracy of data collection. Considering potential conflicts with participants class schedules, this study implemented the intervention in small, staggered groups, and ensured that all participants completed the intervention within each intervention cycle. Participants were grouped and scheduled for training at 14:00 on Mondays, Wednesdays, and Fridays weekly based on their free class times. The specific training protocol (**Figure 2**) was as follows: Each training session began with a standardized warm-up, followed by barbell back squat training. The 8-week training program was divided into three phases: Weeks 1–3 (adaptation phase) at an intensity of 65% 1RM, Weeks 4–6 (improvement phase) with a load of 70% of 1RM, and Weeks 7–8 (intensification phase) with a load of 75% of 1RM. Training was performed 3 times per week, with 3 sets per session and 3-minute inter-set rest intervals, and training volume was controlled within a 20% VL threshold. The 20% VL was selected as the training volume control threshold in this study because existing research indicates that a moderate velocity loss range (> 15% < 30% VL) should be adopted to simultaneously maximize the development of maximal strength, power, and muscle hypertrophy (Jukic et al., 2023); furthermore, studies have confirmed that 20% VL achieves superior comprehensive effects in improving the aforementioned athletic abilities (Pareja-Blanco et al., 2017). Meanwhile, to ensure the individualization and precision of training loads, a linear position transducer was used to monitor the mean concentric MV of each repetition. If a participant’s MV deviated by ± 0.04 m/s from the target range, the load was adjusted promptly to keep MV within the specified zone, and the set was terminated once their VL reached the predetermined threshold; each repetition MV was transmitted in real time to the supporting tablet software via GymAware for real-time monitoring by researchers. During the intervention, all participants received enthusiastic verbal encouragement to complete each repetition with maximum speed and effort, and real-time velocity feedback from the testing system was provided via visual and auditory signals to ensure feedback accuracy and consistency, preventing any impact of feedback bias on training effects (**Table 2**).

**Table 2:** The average number of practice sessions per week per person.

Group	Number of times	Week 1	Week 2	Week 3	Week 4	Week 5	Week 6	Week 7	Week 8
Male	First time	9.81	11.05	11.76	11.29	9.90	9.57	9.94	8.72
	second time	10.76	12.19	10.24	9.76	9.71	9.05	8.67	8.76
Female	First time	10.11	9.58	9.22	11.92	10.44	10.53	9.58	9.64
	second time	10.42	9.89	10.86	10.14	10.11	11.19	9.31	9.69

## 3 Statistical analysis

All data results are presented as mean ± standard deviation, and statistical analysis of the experimental results was performed using SPSS 26.0 (IBM Corp., Armonk, NY, United States). Prior to statistical analysis, the Shapiro-Wilk test for normality and Levene’s test for homogeneity of variances were conducted on the data of each group. Subsequently, repeated-measures analysis of variance (ANOVA) was used to evaluate the effects of the training intervention. The between-group factor was group, including the male and female groups, and the within-subject factor was time, including pre- and post-intervention measurements. This analysis was used to examine the main effects of time and group, as well as the group × time interaction effect. The assumption of sphericity was assessed using Mauchly’s test. When the assumption of sphericity was violated, the Greenhouse–Geisser correction was applied. When significant main effects or interaction effects were detected, *post hoc* pairwise comparisons were performed with Sidak adjustment. All statistical tests were two-tailed, and the significance level was set at *p* < 0.05. The effect size for within-group paired differences was estimated using *Hedges’ g* with 95% CI, with effect size magnitudes classified as follows: trivial effect (*Hedges’ g*≤ 0.2), small effect (0.20 < *Hedges’ g*≤ 0.60), medium effect (0.60 < *Hedges’ g*≤ 1.20), large effect (1.20 < *Hedges’ g* ≤ 2.00), and very large effect (*Hedges’ g* ≥2.0). The effect size for between-group differences in intervention effects was measured using partial eta squared (*ηp²*), with effect size magnitudes categorized as small effect (0.01 ≤*ηp²*≤ 0.06), medium effect (0.06 ≤*ηp²* < 0.14), and large effect (*ηp²* ≥ 0.14) (Aberson, 2010).

## 4 Results and analysis

The changes in each test index before and after the experiment are shown in **Table 3**.

**Table 3:** Changes in each test index before and after the experiment.

Outcome	Group	Pre	Post	Relative change (%)	P	Hedges’g *95% CI*	Time effect			Group effect			Group×time		
							*F*	*P*	*η2 p*	*F*	*P*	*η2 p*	*F*	*P*	*η2 p*
1RM	Male	110.45 ± 18.52	136.36 ± 12.26	23.46	<0.001	1.52 (1.09,3.12)	149.36	<0.001	0.87	31.71	<0.001	0.59	5.61	0.027	0.20
	Female	80.42 ± 12.87	97.92 ± 17.38	21.76	<0.001	1.06 (0.71,1.89)									
CMJ	Male	48.87 ± 6.06	57.52 ± 7.34	17.70	<0.001	1.19 (0.77,2.30)	66.56	<0.001	0.75	47.30	<0.001	0.68	8.61	0.008	0.28
	Female	34.25 ± 6.41	38.32 ± 5.29	11.88	0.001	0.64 (0.27,1.72)									
SJ	Male	47.64 ± 5.55	56.18 ± 6.76	17.93	<0.001	1.28 (0.93,2.35)	125.52	<0.001	0.85	50.93	<0.001	0.70	18.02	<0.001	0.45
	Female	33.59 ± 5.50	37.44 ± 5.22	11.46	<0.001	0.66 (0.36,1.53)									
SLJ	Male	239.91 ± 19.07	251.91 ± 16.28	5.00	<0.001	0.63 (0.51,0.95)	44.97	<0.001	0.67	56.93	<0.001	0.72	6.20	0.021	0.22
	Female	188.75 ± 16.98	194.25 ± 19.29	2.91	0.007	0.28 (0.07,0.65)									
T30	Male	5.31 ± 0.51	4.80 ± 0.27	10.63	0.001	1.15 (0.76,2.16)	58.24	<0.001	0.73	30.78	<0.001	0.58	5.08	0.034	0.19
	Female	6.34 ± 0.54	5.40 ± 0.32	17.41	<0.001	1.95 (1.42,3.33)									
RFT	Male	2.18 ± 0.31	2.34 ± 0.34	7.34	<0.001	0.45 (0.26,0.88)	40.27	<0.001	0.65	3.01	0.097	0.12	0.73	0.40	0.032
	Female	2.00 ± 0.24	2.12 ± 0.24	6.00	<0.001	0.46 (0.25,1.06)									

Positive Hedges’ g values for T30 indicate a reduction in sprint time (improved performance).

### 4.1 Squat 1RM test results

**Figure 3:**
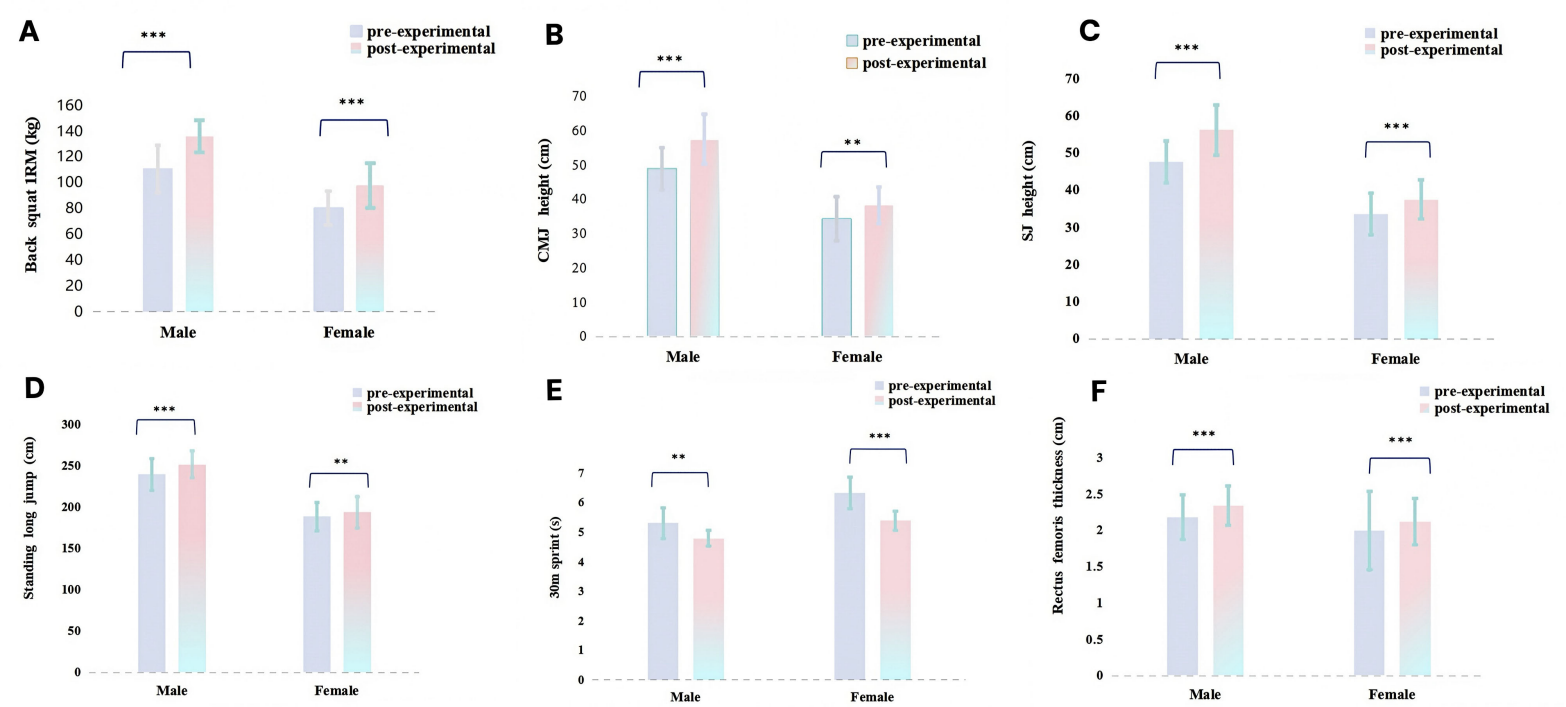
Changes in measured parameters before and after the intervention for the male and female groups. **(A)** Back squat 1RM; **(B)** CMJ Height; **(C)** SJ Height; **(D)** SLJ distance; **(E)** 30m Sprint; **(F)** Rectus Femoris Thickness.

Repeated-measures ANOVA was used to evaluate sex differences in training adaptations of back squat 1RM. The results revealed significant main effects of time (*P* < 0.001) and group (*P* < 0.001), as well as a significant time × group interaction effect (*P* = 0.027) on back squat 1RM. *Post hoc* within-group comparisons indicated that back squat 1RM increased significantly in both the male group (*P* < 0.001, *Hedges’ g* = 1.52) and the female group (*P* < 0.001, *Hedges’ g* = 1.06). *Post hoc* between-group comparisons demonstrated that male athletes exhibited a significantly greater magnitude of improvement in back squat 1RM compared to female athletes (*P* < 0.001) (**Figure 3**).

### 4.2 Jump performance test results

#### 4.2.1 CMJ test results

Repeated-measures ANOVA was used to evaluate sex differences in training adaptations CMJ height. The results revealed significant main effects of time (*P* < 0.001) and group (*P* < 0.001), as well as a significant time × group interaction effect (*P* = 0.008) on CMJ height. *Post hoc* within-group comparisons indicated that CMJ height increased significantly in both the male group (*P* < 0.001, *Hedges’ g* = 1.19) and the female group (*P* = 0.001, *Hedges’ g* = 0.64). *Post hoc* between-group comparisons demonstrated that male athletes exhibited a significantly greater magnitude of improvement in CMJ height compared to female athletes (*P* < 0.001) (**Figure 3**).

#### 4.2.2 SJ test results

Repeated-measures ANOVA was used to evaluate sex differences in training adaptations of SJ height. The results revealed significant main effects of time (*P* < 0.001) and group (*P* < 0.001), as well as a significant time × group interaction effect (*P* < 0.001) on SJ height. *Post hoc* within-group comparisons indicated that SJ height increased significantly in both the male group (*P* < 0.001, *Hedges’ g* = 1.28) and the female group (*P* < 0.001, *Hedges’ g* = 0.66). *Post hoc* between-group comparisons demonstrated that male athletes exhibited a significantly greater magnitude of improvement in SJ height compared to female athletes (*P* < 0.001) (**Figure 3**).

#### 4.2.3 SLJ test results

Repeated-measures ANOVA was used to evaluate sex differences in training adaptations of SLJ performance. The results revealed significant main effects of time (*P* < 0.001) and group (*P* < 0.001), as well as a significant time × group interaction effect (*P* = 0.021) on SLJ performance. *Post hoc* within-group comparisons indicated that SLJ performance improved significantly in both the male group (*P* < 0.001, *Hedges’ g* = 0.63) and the female group (*P* = 0.007, *Hedges’ g* = 0.28). *Post hoc* between-group comparisons demonstrated that male athletes exhibited a significantly greater magnitude of improvement in SLJ performance compared to female athletes (*P* < 0.001) (**Figure 3**).

### 4.3 Short-distance sprint performance test results

Repeated-measures ANOVA was used to evaluate sex differences in training adaptations of T30 performance. The results revealed significant main effects of time (*P* < 0.001) and group (*P* < 0.001), as well as a significant time × group interaction effect (*P* = 0.034) on T30 performance. *Post hoc* within-group comparisons indicated that T30 performance improved significantly in both the male group (*P* = 0.001, *Hedges’ g* = 1.15) and the female group (*P* < 0.001, *Hedges’ g* = 1.95). *Post hoc* between-group comparisons demonstrated that female athletes exhibited a significantly greater magnitude of improvement in T30 performance compared to male athletes (*P* < 0.001)(**Figure 3**).

### 4.4 RFT test results

Repeated-measures ANOVA was used to evaluate sex differences in training adaptations of RFT. The results revealed that only the main effect of time reached statistical significance (*P* < 0.001), while neither the main effect of group (*P* = 0.097) nor the time × group interaction effect (*P* = 0.40) was statistically significant. *Post hoc* within-group comparisons indicated that RFT increased significantly in both the male group (*P* < 0.001, *Hedges’ g* = 0.45) and the female group (*P* < 0.001, *Hedges’ g* = 0.46). *Post hoc* between-group comparisons showed no significant difference in the magnitude of RFT improvement between male and female athletes (*P* = 0.097) (**Figure 3**).

## 5 Discussion

This study examined sex-related differences in adaptations to velocity-based resistance training among male and female athletes with matched relative strength levels with equivalent baseline relative strength. The outcomes included maximal lower-limb strength, explosive performance, and muscle thickness. Three primary outcomes were identified. First, after 8 weeks of squat-based VBT with a 20% VL threshold, both male and female athletes showed improvements in maximal lower-limb strength, jumping ability, T30 performance, and rectus femoris thickness. Second, sex differences were mainly observed in measures of maximal strength and explosive performance. Males exhibited greater gains in 1RM, CMJ, SJ, and SLJ, while females achieved superior improvements in the T30. Finally, although RFT increased significantly after training, no significant sex difference was observed.

The results showed that both male and female athletes achieved significant improvements in maximal strength, explosive performance, and muscle thickness after 8 weeks of VBT. These findings align with prior investigations (Rossi et al., 2024; Han et al., 2025; Lin et al., 2025). Among the performance outcomes, the improvement in T30 was particularly notable, especially in female athletes. This result may be related to the positive transfer of back squat-based VBT to sprint acceleration ability. The back squat can enhance lower-limb force production, particularly the strength of the hip and knee extensors. Lower-limb strength and the ability to apply horizontal force during acceleration are both closely related to sprint performance (Morin et al., 2012; Seitz et al., 2014). In addition, VBT may further promote rapid force production and neuromuscular coordination, thereby contributing to the improvement in T30 performance. The greater improvement in the female group may be partly explained by their lower baseline T30 performance, which allowed greater room for improvement. However, the proposed mechanism for the transfer of VBT to sprint performance remains speculative, and the causal relationship needs to be verified in future studies.

Overall, these improvements in performance may be mainly attributed to the ability of VBT to control training loads more precisely. VBT is less affected by individual physiological fluctuations and can produce similar or even greater improvements in performance with lower levels of fatigue (Liao et al., 2021; Weakley et al., 2021). Notably, beyond these general adaptations, we also observed several interesting and important findings.

Regarding the development of maximal strength and explosive performance, our study found that after 8 weeks of VBT, the male group showed larger relative gains and higher effect sizes in squat 1RM and jump-related measures than the female group. In contrast, the female group showed greater positive adaptation in the T30 test and a greater effect size. Notably, these findings are not consistent with those reported in previous studies (Ramirez-Campillo et al., 2016; Rissanen et al., 2022; Lecce et al., 2025).

In previous studies on maximal strength development, Rissanen et al. implemented an 8-week, twice-weekly training intervention and found no significant sex differences in squat 1RM gains under a 20% velocity loss threshold (Rissanen et al., 2022). Similarly, Lecce et al. recruited 10 male and 10 female elite athletes for a 4-week squat training intervention. Their results showed that squat 1RM increased in elite athletes across all velocity loss conditions, with no significant sex differences in strength gains (Lecce et al., 2025). Similar findings have also been reported for vertical jump and sprint performance. Rissanen et al. found that both men and women improved their CMJ performance after 8 weeks of VBT. However, no statistically significant difference was observed between sexes. Ramírez-Campillo et al. reported that after 6 weeks of plyometric training, both male and female soccer players improved their jumping ability, T30, change-of-direction speed, and endurance. However, the magnitude of training adaptation did not differ significantly between sexes (Ramirez-Campillo et al., 2016). Overall, previous evidence suggests that males and females exhibit comparable training adaptations to similar training interventions. This appears to apply not only to maximal strength, but also to vertical jump and sprint performance.

The discrepancies described above may be related to differences in baseline fitness, training background, and intervention modalities. In the study by Rissanen et al., the baseline 1RM/BM was significantly lower in women (1.00) than in men (1.38), which may have afforded females greater adaptive potential (Rissanen et al., 2022). In contrast, in the present study, the male and female groups had similar baseline relative strength levels. This may have made sex-specific adaptations more apparent. In addition, the participants in the study by Lecce et al. were international-level elite athletes (Lecce et al., 2025), whereas the participants in the present study were collegiate athletes. In high-level athletes, neuromuscular adaptations may already be close to their upper limit, leaving limited room for further improvement and heightening the occurrence of training plateau (Pareja-Blanco et al., 2017). By contrast, collegiate athletes may still have greater adaptive potential. Therefore, potential sex-related differences in strength development may be easier to detect in this population. Finally, the present study used VBT, whereas Ramírez-Campillo et al. used plyometric training (Ramirez-Campillo et al., 2016). Plyometric training has been shown to induce similar performance improvements in male and female athletes (Ramirez-Campillo et al., 2023). Because the underlying mechanisms of these interventions differ, VBT inherently emphasizes loaded movement velocity, force–velocity characteristics, and individualized load prescription. These characteristics may make sex-specific adaptations in certain performance outcomes more observable. Therefore, in populations with similar baseline relative strength but relatively lower training status, the effects of VBT on maximal strength and explosive performance may show a degree of sex specificity.

While both male and female participants showed increases in rectus femoris thickness following the intervention, there was no statistically significant difference in the magnitude of change between groups. This suggests that this training method produced similar changes in rectus femoris thickness in males and females. Andersen et al. conducted a 9-week velocity-based resistance training intervention in healthy men and women. The intervention included leg press and seated leg extension exercises with either a low VL threshold (15% VL) or a high VL threshold (30% VL). They found that, when training volume was matched, the two conditions produced similar effects on maximal muscle hypertrophy, in addition to similar muscle adaptations (Andersen et al., 2024). Similarly, Kojić et al. conducted a 7-week squat resistance training intervention, performed twice per week, in male and female college students without prior resistance training experience. Their results showed no significant sex differences in changes in quadriceps muscle cross-sectional area (Kojić et al., 2021). In addition, a recent review reported that, after adjusting for baseline differences between men and women before training, resistance training-induced increases in muscle cross-sectional area were similar between sexes (Lecce et al., 2026). Taken together, these findings suggest that, under comparable training volume and training protocols, resistance training has generally similar effects on hypertrophy-related outcomes in men and women. Sex may not be a major determinant of differences in this type of training adaptation.

While our study has yielded the above novel findings, it also has several limitations. First, physiological indicators during training (e.g., blood lactate, heart rate variability, electromyography) were not simultaneously monitored, which prevents us from further elucidating the mechanisms underlying sex differences from the multi-dimensional “metabolic-neuromuscular” perspective. For example, we could not determine whether the improvement in female sprint performance is associated with sympathetic nervous system regulatory efficiency. Second, although our study analyzed the effects of training on performance in male and female athletes under specific load intensities and velocity loss thresholds, it did not comprehensively investigate sex differences in performance across varying load intensities and velocity loss thresholds. This limitation restricts our ability to conduct a comprehensive assessment of how training efficacy varies with changes in training conditions. Third, although female participants were tested outside the menstrual bleeding phase before and after the intervention, menstrual cycle fluctuations during the 8-week training period were not fully controlled. These fluctuations may have influenced training status, recovery, and exercise motivation. Future studies should monitor or control menstrual cycle phases throughout the intervention period. Finally, we did not record the number of load adjustments throughout the intervention or the sex difference in adjustment frequency. The absence of this data is a limitation of this study.

## 6 Conclusion

The present study found that, under similar baseline relative strength levels, both male and female athletes showed significant improvements in maximal lower-limb strength, explosive performance, and rectus femoris thickness after 8 weeks of barbell squat VBT using a 20% VL threshold. However, the effects of VBT on maximal strength and explosive performance may show a degree of sex specificity, whereas its effects on hypertrophy-related outcomes, such as rectus femoris thickness, appear to be relatively consistent between sexes. Therefore, in practical training, VBT programs should be designed with greater specificity by considering sex, training status, and sport-specific demands, while maintaining comparable training loads and protocols.

## Data Availability

The raw data supporting the conclusions of this article will be made available by the authors, without undue reservation.
